# Etymologia: *Pseudoterranova decipiens*

**DOI:** 10.3201/eid2811.220792

**Published:** 2022-11

**Authors:** William C. Partin, Richard S. Bradbury

**Affiliations:** Emory University, Atlanta, Georgia, USA (W.C. Partin);; Federal University of Australia, Berwick, Victoria, Australia (R.S. Bradbury)

**Keywords:** Pseudoterranova decipiens, nematode, parasites, Terra Nova ship, Edward Leicester Atkinson, Robert T. Leiper, Aleksei Mozgovoi, Captain Robert Scott

## [sü-dō-′ter-ə-nō-və-a dee-sip′-e-inns]

**Figure 1:**
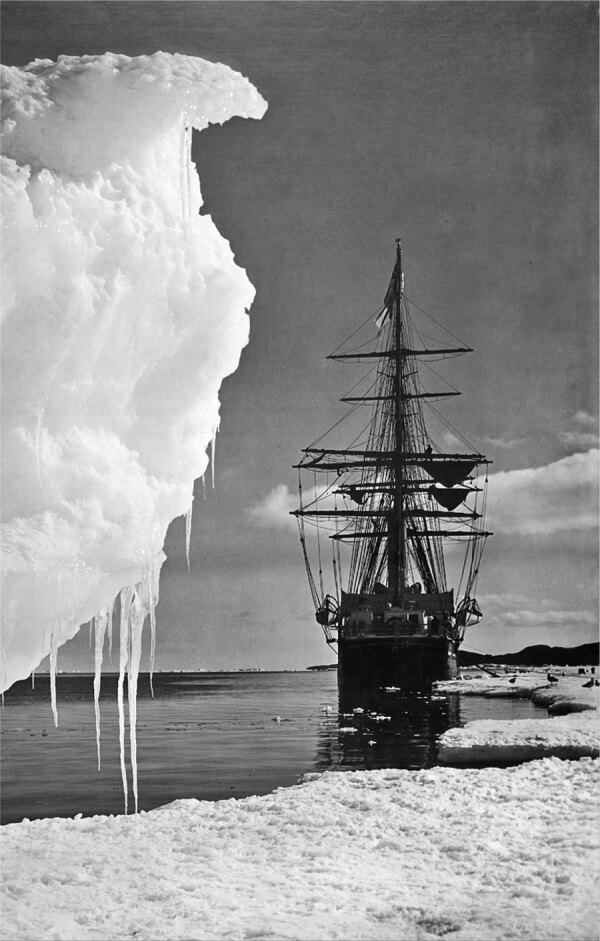
The Terra Nova, 1911 (1937). Captain Robert Falcon Scott's (1868–1912) ship the Terra Nova in the Antarctic on the ill-fated expedition to the South Pole. A print from The Story of Seventy Momentous Years, the Life and Times of King George V, 1865–1936, editor Harold Wheeler, Odhams Press Ltd, London, 1937. The *Terra Nova* at the ice edge in Antarctica.

The report of 12 South Korean persons infected with *Pseudoterranova decipiens,* described in a recent issue of Emerging Infectious Diseases, has prompted the editors to revise the errant course charted in 2011 for this genus. By failing to mention the ship *Terra Nova*, a nomenclatural oversight occurred. A compelling but overlooked nautical provenance for the etymology of this genus existed, which prompted Scott Norton of Georgetown University and David Gibson from the Natural History Museum in London, to gently and cleverly advise that “someone literally missed the boat.” *Terra Nova* is easily translated as “New Earth,” “New Land,” or even “Newfoundland.” The *Terra Nova,* a whaling ship, was refitted for the British Antarctic Expedition. Under the command of Captain Robert Scott, the *Terra Nova* departed from Wales in 1910 (Figure 1). The main purpose of the expedition was to achieve primacy for attaining the South Pole and was akin to discovering new land, perhaps a symbolic affirmation for the name of their sailing vessel. As Scott and others pursued their goal, ship surgeon Edward Leicester Atkinson remained on board, capturing and dissecting marine life. While doing so, he discovered an “unusual nematode” infesting a shark. In 1914, Atkinson, and London School of Tropical Medicine parasitologist Robert Thomson Leiper named this nematode Terranova Antarctica in honor of the RRS Terra Nova.

**Figure 2:**
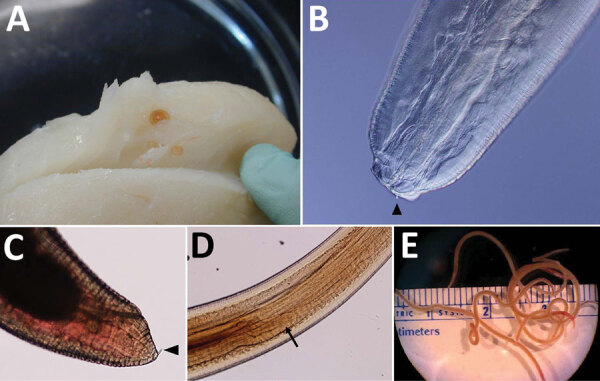
A) A coiled *Pseudoterranova* sp. L3 larva in a fillet of cod. B) View of the anterior (head) end of an aniskaid larvae, possibly *Pseudoterranova* sp*.,* showing lips and an indistinct boring tooth (arrowhead) viewed by differential interference contrast microscopy. C) posterior with mucron (arrow). D) Center (cleared with lactophenol) demonstrating the ventriculus and anteriorly directed intestinal cecum (arrowhead). E) gross morphology of adult *Pseudoterranova* sp. L3 larvae. Original magnifications ×100 in panels B, C, and D; ×10 in panel E. Blaine Mathison, Henry Bishop, Division of Parasitic Diseases, Centers for Disease Control and Prevention.

The genus *Pseudoterranova* was first proposed by Aleksei Mozgovoi in his 1950 unpublished thesis. The genus name was introduced in multiple papers and book chapters in 1951, although which publication has primacy is debated because later researchers differed in their designated attributions. The type species was the former *Porrocecum kogiae* (syn. *Terranova kogiae*), as this helminth, taken from a South Australian pygmy sperm whale (*Kogia bereviceps*), was morphologically distinct from both the genera *Terranova* and *Porrocecum.* The genus Terranova still exists, but is restricted only to parasites of elasmobranch fish (Figure 2).

The species epithet *decipiens* was applied to *Ascaris decipiens* by Krabbe in 1878. The word *decipiens* is a Latin third declension participle. The primary meaning attached to this word is to catch, take, ensnare, or seize with a secondary meaning to cheat, deceive, beguile, or mislead. Although Krabbe did not state his reasons for applying this name, it seems likely to have been in reference the catching or taking of fish by the seal hosts from which he first recovered the worm.

Over the following 105 years, this species was moved between the genera *Porrocecum*, *Terranova*, and *Phocanema*, before finally being placed in the genus *Pseudoterranova* by Gibson in 1983. Molecular interrogation later demonstrated that there is a robust *P. decipiens* species complex, incorporating 5 sibling species, including *P. decipiens sensu stricto.*

The fate of the *Terra Nova* and Captain Scott’s expedition was forlorn. After reaching the South Pole, Scott and 4 other explorers, their supplies exhausted, perished while on the return trek to the *Terra Nova*. The doomed party was disappointed to discover that Roald Amundsen and his group had preceded them to the South Pole by 34 days. The *Terra Nova* continued an active seafaring life in various capacities. In 1943, near the coast of Greenland, while ferrying supplies for the US government, she sank after ramming into ice floes. All aboard were rescued by the USCGC Atak. The Terra Nova was purposefully set afire and then sunk by gunfire to eliminate it as a shipping lane hazard. Exactly a century after Scott’s team reached the South Pole, a scientific research vessel in 2012 serendipitously discovered the *Terra Nova* resting on the ocean floor.
